# Detecting Positioning Errors and Estimating Correct Positions by Moving Window

**DOI:** 10.1371/journal.pone.0143618

**Published:** 2015-12-01

**Authors:** Ha Yoon Song, Jun Seok Lee

**Affiliations:** Department of Computer Engineering, Hongik University, Seoul, Korea; Beijing University of Posts and Telecommunications, CHINA

## Abstract

In recent times, improvements in smart mobile devices have led to new functionalities related to their embedded positioning abilities. Many related applications that use positioning data have been introduced and are widely being used. However, the positioning data acquired by such devices are prone to erroneous values caused by environmental factors. In this research, a detection algorithm is implemented to detect erroneous data over a continuous positioning data set with several options. Our algorithm is based on a moving window for speed values derived by consecutive positioning data. Both the moving average of the speed and standard deviation in a moving window compose a moving significant interval at a given time, which is utilized to detect erroneous positioning data along with other parameters by checking the newly obtained speed value. In order to fulfill the designated operation, we need to examine the physical parameters and also determine the parameters for the moving windows. Along with the detection of erroneous speed data, estimations of correct positioning are presented. The proposed algorithm first estimates the speed, and then the correct positions. In addition, it removes the effect of errors on the moving window statistics in order to maintain accuracy. Experimental verifications based on our algorithm are presented in various ways. We hope that our approach can help other researchers with regard to positioning applications and human mobility research.

## Introduction

**Fig 1:**
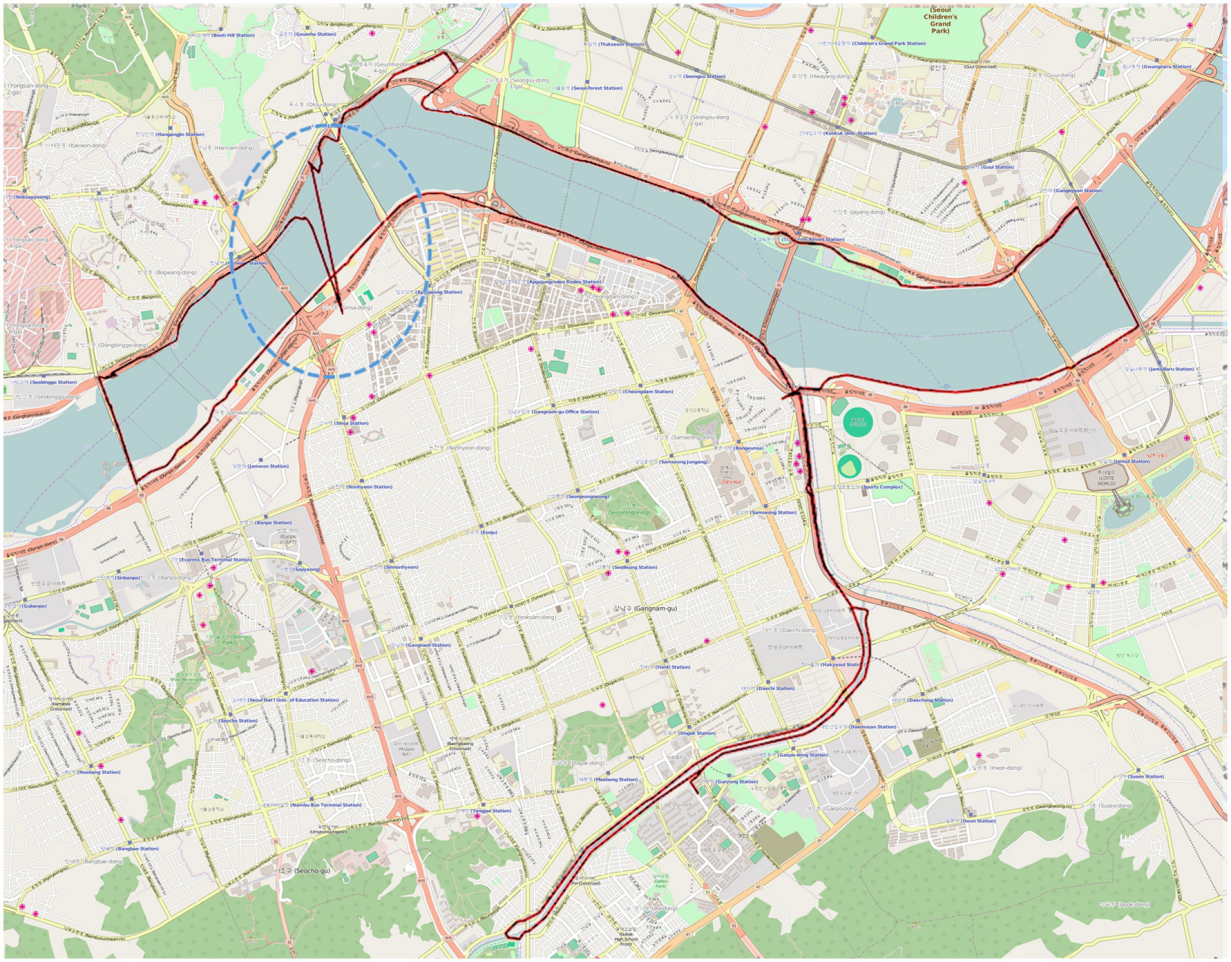
Positioning Error in an Actual Trajectory.

Many portable smart devices, such as smartphones or Global Positioning System (GPS) receivers, allow location-based and other related services. The core functionality of such services is the positioning ability of mobile devices. Recent mobile devices have positioning abilities that are based on GPS, Global Navigation Satellite System (GLONASS), Galileo, satellite-based positioning systems, and other terrestrial-based positioning systems. Cellular station-based positioning and crowd-sourced Wi-Fi positioning methods are currently being used. The main problem addressed in this paper is the positioning error in such systems. All positioning systems have errors mainly caused by environmental factors. The positioning errors in mobile devices are sometimes critical and can degrade the quality of services that are based on the positioning functionality. Considering this, detecting positioning errors and eliminating error values is desirable, especially when the error can be calibrated within a tolerable range or can be corrected to a plausible position. However, singular positioning errors cannot be detected as there is no indication as to whether a single positioning data is indeed an error. Nevertheless, errors that have a typical peak can be detected from the correct data set. For example, Fig 1 shows errors in a bicycle run trajectory on a map. A positioning device, Garmin EDGE 500 [1], which is a GPS receiver, was used to obtain the positioning data set. The yellow line shows the trajectory of the positioning data collected by Garmin EDGE 500. This bicycle run trajectory shows an erroneous trajectory near the big bridge due to environmental errors. A part of trajectory in the red oval abruptly goes across the river without crossing the bridge and then returns to the normal trajectory.

**Fig 2:**
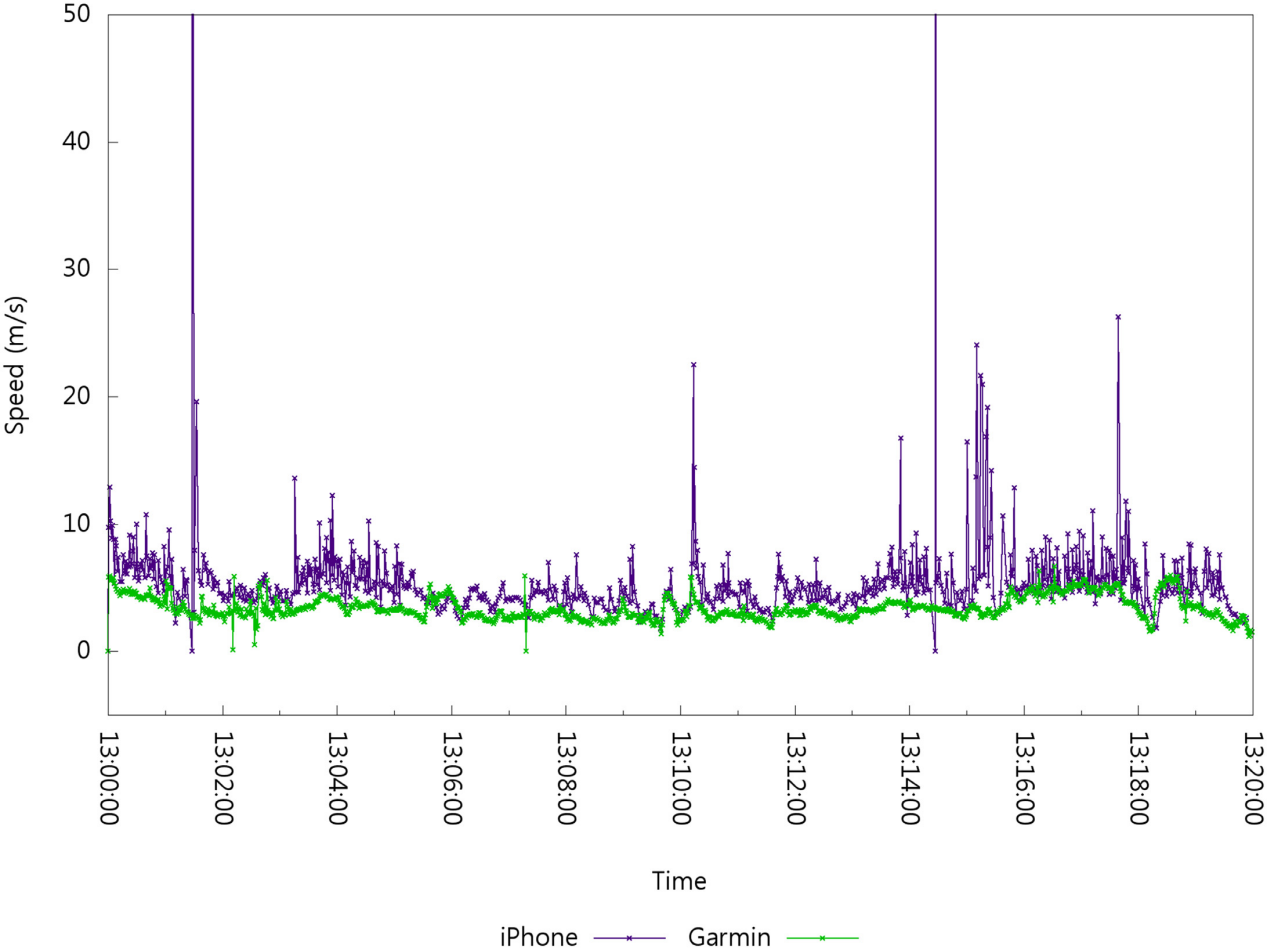
Speed Error in the Positioning Data Set.

It is also possible to calculate the speed between two consecutive positioning data. The distance between two positioning data can be calculated; this distance can be used, along with the time difference of two consecutive positioning data, to calculate the speed. Fig 2 shows the speed values calculated from the positioning data set by Garmin EDGE 800 [2] and iPhone 4S [3]. The x-axis shows the time of the day and y-axis shows the corresponding speed value.

Fig 2 also shows abnormal speed values due to positioning errors. For example, the maximum speed measured by the iPhone 4S is 291.028 m/s with an average speed of 6.16464 m/s from the 763 data collected, whereas the maximum speed measured by EDGE 800 is 6.65531 m/s with an average speed of 3.39092 m/s from the 1200 data collected. The difference in value between the two devices shows the effect of error in the positioning system, especially in a mobile state. Positioning errors imply speed errors; thus, we focus on speed errors as clues for detecting positioning errors. Similar to speed values, the acceleration values between two consecutive speeds can be calculated. Once abnormal speed or acceleration values are detected, positioning errors can be identified. The detection of unreal or abnormal values can cause another problem. The term *unreal value* refers to physically unreachable values such as 1000 m/s on Earth. We define the term *abnormal values* as extraordinary when compared with neighboring values. In order to detect unreal or abnormal values, the concept of moving window velocity values over time domain is introduced.

Our purpose is to detect and eliminate such erroneous positions or estimate correct positions on mobile devices. Because of the nature of mobile devices, such an algorithm must cope with minimal computational power and memory, and limited battery capacity. Our algorithm is clearly a part of the outlier detection problem and requires a time series analysis method.

## Background and Related Research

Positioning devices receive signals from positioning system components and determine the position at a given time. It is well known that a positioning system always has the potential of determining erroneous positions caused by environmental errors. The outline of up-to-data positioning technologies must be presented in order to understand this research, as well as the method for speed calculation using positioning data.

### Positioning Technologies

The most popular positioning systems currently in operation or soon to be deployed are GPS [4], GLONASS, and Galileo [5].

In addition to satellite-based positioning systems such as GPS and GLONASS, there are indoor positioning systems such as Wi-Fi fingerprinting. The latter can identify the position of a device inside a building where the device receives radio signals from a Wi-Fi Access Point (AP) and calculates its location. The fundamental principle behind this mechanism is that the signal strength received by the device is the reciprocal of the log of the distance between the device and AP [6].

A similar mechanism based on the strength of the Bluetooth signal, known as Bluetooth-based positioning, also exists [7]. The system is based on the empirical product of the Bluetooth signal strength and distance.

A cellular station-based positioning system utilizes the Time Difference of Arrival (TDOA) to measure the position of a mobile phone. A previous cellular station-based positioning system was based on simple TDOA and was dependent on the events initiated by the mobile phone user. A Scheduling and Tracking System (STS) later appeared with frequent updates of one or more designated cellular phone positions [8]; however, it is now slightly outdated technology.

There are hybrid positioning systems that combine all possible positioning systems. For example, a combination of the Satellite-based Positioning System (SPS) and Wireless Local Area Network (WLAN)-based Positioning system (WPS or WLANPS) was developed by Skyhook and it is known as the Skyhook Positioning System [9]. This system successfully avoids the urban canyon phenomenon by additionally utilizing WLANPS. A minimum of two satellites are employed to identify the approximate WLAN AP positions, and these WLAN APs can be used as APs for Wi-Fi fingerprinting.

Currently, it is possible for everybody to carry positioning devices such as GPS receivers or smartphones with positioning functionalities. In this research, we collected positioning data from as many devices as possible. Positioning data sets were collected by several volunteers starting from November 2011. Most volunteers collected such positioning data using smartphones with positioning capabilities such as iPhone 4 [10], iPhone 4s [3], Galaxy S3 [11], Galaxy Note 2 [12] and iPhone 5s [13]. Some volunteers carried additional GPS receivers such as Garmin EDGE 800 [2], Garmin GPSMAP 62s [14] and Garmin EDGE 810 [15] to collect the positioning data.

### Speed Calculation using Positioning Data

The positioning data obtained by a positioning system is usually in a triplicate form that contains longitude, latitude, and time. We refer to this trio as the positioning tuple because tuples have more informative attributes. Once we obtain two consecutive positioning tuples, it is possible to calculate the speed between such positions. Because Earth is an oblate ellipsoid, several methods have been developed for such calculations.

The haversine formula is used to calculate the distance between two points from two pairs of latitude and longitude values [16]. This simple method assumes that Earth is a sphere and calculates the distance between two points using the Earth’s radius. Each location has its own radius-factor on the Earth’s surface because the Earth’s radius depends upon a specific location on the Earth’s surface. Therefore, this formula is slightly erroneous for calculating large distances because it assumes that Earth is a sphere instead of an oblate ellipsoid.

The more accurate and elaborate method must assume that Earth is an ellipsoid. For an oblate ellipsoid, the surface between any two position points is curved and therefore the distance of the shortest curved path is calculated. The equilateral radius of Earth and the polar semi-axis are used to calculate the flattening and eccentricity and also to identify the ellipsoid shape of Earth. As shown in [17], the distance is calculated up to a 2,000 Km range, and in 2011, this method is successful to calculate distance to the unit in nanometers [18].

We use the haversine formula in our research, which is simpler than other methods, because we collect positioning data over relatively short distances. The maximum calculable distance in this research is 300 m, thus the haversine formula can be used in our research.

### Positioning and Speed Errors

Most positioning systems can have positioning errors. For example, satellite-based positioning systems are very accurate in outdoor situations, but they have larger errors in indoor settings because of the line-of-sight problem. Table 1 lists typical results for error rates and other statistics for GPS and cellular-based station positioning systems obtained through basic experiments. The notation n(⋅) denotes the count, E[⋅] is the expectation, Max(⋅) is the maximum value, and *σ* is the standard deviation.

**Table 1:** Typical Errors during Positioning Data Acquisition.

**Location**	**Cellular Base Station**		**GPS**	
Indoor	n(Data Point)	893	n(Data Point)	2186
	n(Error Point)	434	n(Error Point)	939
	Error Rate	48.6%	Error Rate	43.0%
	E[Error Dist]	52.5530m	E[Error Dist]	43.5506m
	Max(Error Dist)	156.7578m	Max(Error Dist)	10769.72m
	*σ*_*ErrorDist*_	32.6859m	*σ*_*ErrorDist*_	370.6034m
Outdoor	n(Data Point)	331	n(Data Point)	1690
	n(Error Point)	122	n(Error Point)	208
	Error Rate	36.9%	Error Rate	12.3%
	E[Error Dist]	52.6618m	E[Error Dist]	4.4498m
	Max(Error Dist)	206.3526m	Max(Error Dist)	51.7789m
	*σ*_*ErrorDist*_	23.5953m	*σ*_*ErrorDist*_	7.1696m

It is usually assumed that a stationary device, i.e. a device that does not move, acquires a more precise position than a mobile device. From two consecutive positions and timing information, the distance and speed between the two consecutive positions can be easily calculated. A positioning error implies that the calculated speed value is abnormal. Once an abnormal speed value is found, the positioning data that produced the abnormal speed values are considered to correspond to the erroneous positions. Similar phenomena can occur with abnormal acceleration values. Once unusual acceleration values are found, the positioning data corresponding to these acceleration values are considered to be erroneous. Table 2 lists the maximum possible speeds for different transportation methods; the values in Table 2 can be used as criteria to determine abnormal speed values.

**Table 2:** Maximum Speed for different Transportation Methods.

**Transportation Method**	**Maximum Speed (*m*/*sec*)**
Ambulation	3.00
Bicycle	33.33
Automobile	92.78
Sports-Car	244.44
High-Speed Train	159.67
Airplane	528.00

Our research assumes a continuous input of positioning data sequences using positioning devices such as smartphones or GPS receivers. Positioning devices can acquire position data every second or when they sense a change in position, i.e., detect movement. This situation requires the Time Series Analysis (TSA) concept because detecting erroneous positioning data is a type of an outlier detection problem.

Because our purpose is to detect positioning data with abnormal speed values when compared with the tendency of the neighboring speed values, the concept of a moving window is introduced. A moving window contains several positioning data at a given time, and several useful statistics can be calculated from the data in the window. Among the various moving window statistics, the Moving Average (MA) and Moving Standard Deviation (MSD) are considered in this research. The concept of MA is used widely in various research areas, whereas the concept of MSD is seldom used. The combination of MA and MSD is the basis of our research. Detailed ideas are discussed in the following section, Idea of a Moving Window.

### Minimizing Positioning Error

Lengthy trials have been conducted in order to improve the positioning data accuracy. The GPS system is the best positioning system as it has various mechanisms to improve the accuracy of the positioning data. Most GPS-related improvements consider underlying hardware systems and tend to extend hardware schemes [19]. In addition to hardware extensions, postprocessing of the GPS data [20] is another method which does not require real-time processing.

A software approach that does not consider the underlying positioning system and assumes that a mobile device is used has been developed by one of the authors of this paper. In 2012, initial trials were conducted and an algorithm for detecting the speed error was developed based on the moving window [21]. Following this, a trial to estimate the correct data corresponding to the erroneous positioning data was presented [22]. However, these two studies incorrectly assume that human mobile speed follows a normal distribution.

Results of recent studies indicate that probability distributions, excluding normal distributions, are well fit for human mobile speed [23, 24], [25]. Therefore, the core part of the algorithm must be developed again, along with other options for positioning error detection and correction.

Other areas of research that combine a positioning system and moving window can be found in a genetic algorithm [26], and for meteorology applications [27] but they are extremely rare.

## Idea of a Moving Window

Because of the nature of our problem, a sequence of speed values must be utilized and temporal trends should be found. Because one of the requirements is low computing power and memory capacity, autoregressive, exponential, and quadratic methods are eliminated from the candidate list. A linear approach is selected for trend modeling. To smooth the varying speed values, an MA approach, rather than exponential smoothing, is selected. For the research on MA, a detailed method has been described in [28], whereas our algorithm requires real-time processing on mobile devices.

Our method must detect abnormal values to be out-of-trend values. For example, if the data shows that a speed of 100Km/hr is achieved while driving at approximately 30Km/hr, the speed value is clearly an abnormal value. It has been known that such positioning errors are Circular Error Probable (CEP) based on a circular bivariate normal distribution, and the speed errors derived from positioning errors need to be identified. Several possible candidates for human mobile speed distributions can be found in [23]. Among the candidate distributions, the exponential distribution is chosen because of its ease of calculation.

In order to determine tendency of speed, a moving window is introduced. It contains several most recent positioning tuples that contain timestamp and position information. If a moving window contains *n* most recent tuples, the size of the moving window is *n*.

As already discussed, speed and acceleration values can be calculated from consecutive tuples, and these values are also a part of members of the positioning tuple. From the sequence of speed values in a moving window, we can construct statistics such as speed MA and MSD values. From these MA and MSD values, it is possible to determine the parameters for exponential distribution, and the scale and location. The MSD value itself is converted directly into a scale parameter, and the difference between the MA and MSD values gives the location parameter.

It is also possible to calculate the latitude and longitude variation at a given time with consecutive tuples. Thus, the MA and MSD values of the latitude and longitude variation can also be members of the positioning tuples.

With these MA and MSD values, we can construct a significant interval with a given significant level *s*. The significant interval constructed by the moving window statistics at a given time is referred to as the Moving Significant Interval (MSI).

We then have the parameters to obtain the human mobile speed distribution at a given time. With a user-defined significant level *s*, the MSI size can be determined. From the theory of probability distribution, we can detect abnormal speed values under any given rate controlled by *s*.

On this basis, the velocity value of MSI can be detected, filtered, or corrected using the user-defined options and parameters.

## Considerations on Environments and Parameters

### Environments

Every positioning system has errors that occur due to various sources. The most distinguishing positioning systems in the world are GPS, GLONASS, Galileo, cellular base station-based positioning, crowd source Wi-Fi positioning, and a hybrid of several of these systems. Among these positioning systems, the positioning errors in GPS systems have been measured and researched through various methods. Table 3 shows an analysis of the error sources. Because of the existence of many tall buildings in downtown areas, known as urban canyons, the error rates and volumes are higher than those in country areas. A GPS accuracy report [29] summarized the accuracy issues of GPS systems. Several variants of GPS systems have been developed to increase the utilization and accuracy of the original GPS system. Table 4 lists the variants of GPS systems with typical distance errors. Our algorithm characterizes the typical errors of a positioning system with the parameter *ET*_*D*_, which represents the error tolerance of distance from the inherited distance error. This is a user-defined parameter and it can be reset when the error distance of the positioning system is known.

**Table 3:** Typical Error Sources in GPS Systems.

**Source**	**Error in distance (meters)**
Inospheric effects	±5
Shifts in satellite orbits	±2.5
Clock errors of satellite clocks	±2
Multipath effects	±1
Tropospheric effects	±0.5
Calculation and rounding errors	±1

**Table 4:** Typical Distance Errors in GPS System.

**GPS variants**	**Accuracy (meters)**
GPS system with SA activated	±100
SA deactivated	±15
Differential GPS	±3 − 5
with WAAS/EGNOS	±1 − 3

### User-Defined Parameters

Positioning data set error rates have been obtained from previous experiments. The error rate of the positioning data set depends on the positioning system, positioning environment, and the capability of the positioning receiver. As shown in Fig 2, a dedicated Garmin GPS receiver performs better than the iPhone, a multipurpose smartphone, for the same route, time, and date. Thus, a user is required to predetermine the Significant Levels (SLs) that designate the parameters for determining the significant interval size, which is a popular method in normal distribution. Because speed value is always positive, only the upper half of the significant interval is used. A user can control the sensitivity of the predicted error rate by statistically controlling *s*.

By combining the error tolerance of the positioning system, *ET*_*D*_, and the possible minimum speed of human ambulation, MINSPEED, the minimum speed for our algorithm can be determined. The minimum speed, *MIN*_*speed*_, for our algorithm is the maximum speed determined by *ET*_*D*_ and MINSPEED, which are user-defined parameters, and both are required for the execution of our algorithm. The parameter *MIN*_*speed*_ is also used to guarantee the minimum required length of MSI.

Similar to *MINSPEED*, a phenomenal and natural parameter for human ambulation, *MAX*_*acceleration*_, is required for our algorithm. This is the maximum possible acceleration value of an object’s mobility on Earth. Once the calculated acceleration value for a positioning tuple exeeds *MAX*_*acceleration*_, the tuple is instantly regarded as erroneous. Both *MINSPEED* and *MAX*_*acceleration*_ can be hardwired in the algorithm. For our experiment, the values chosen were *MINSPEED* = 2.0*m*/*s* and *MAX*_*acceleration*_ = 10.8*m*/*s* ^2^.

Another user-defined parameter is the window size. The upper and lower limits of the window size are required to be known because our algorithm adjusts the window size according to the number of continuous errors. The parameters related to the window size are discussed in the following subsection.

### Window Size

We need to determine the appropriate window size. A small window size is a good fit for devices with small memory capacity. It can be easily assumed that a smaller window size can cope with abrupt changes in speed; however, it is prone to being affected by speed errors. If a small window size is selected, the occurrence of large speed error values affects the MA and MSD values significantly. Conversely, a big window size leads to a tendency of tailing effects, and hinders the speed change from being reflected in the window statistics. The newly obtained positioning tuples with relatively higher speed inside the moving window then have a high possibility of being filtered. Here we define the terms *overfiltering* and *underfiltering*. Overfiltering is a phenomenon in which a tuple with correct values is filtered. This usually occurs with a monotone increase in the speed because the moving window statistics remain unchanged because they are calculated with past speed values. Underfiltering is a phenomenon in which a tuple with incorrect values is not filtered. In this case, a possible speed error cannot be filtered because of the large values of the past moving window statistics. Overfiltering and underfiltering are always possible because future speed values cannot be predicted and only current moving window statistics are known.

**Fig 3:**
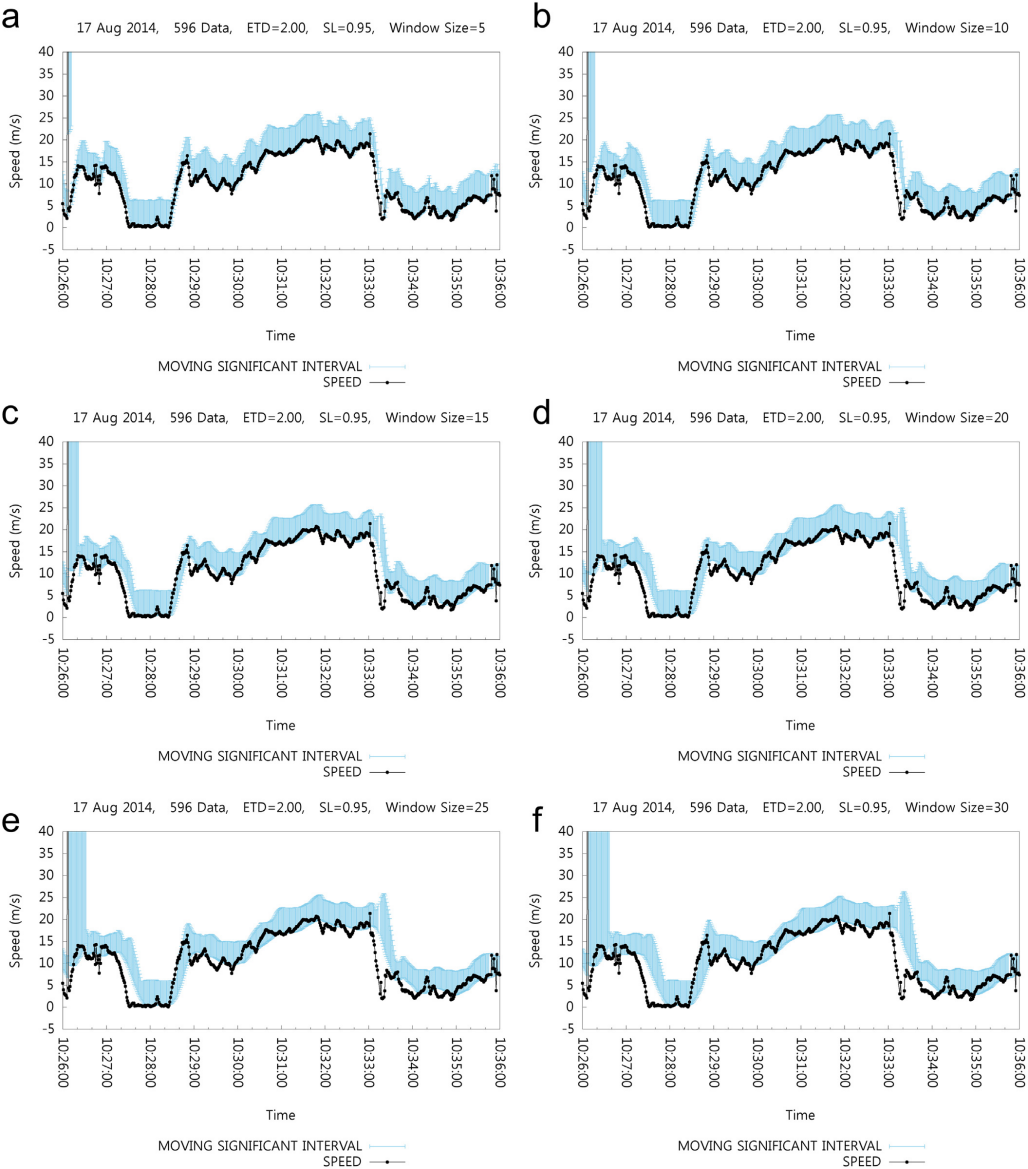
Effect of Various Window Sizes.

Therefore, we conducted a preliminary experiment on the effect of window size on the performance of our algorithm. Fig 3 shows the effect of window size on the moving window and moving window statistics. The x-axis represents time of day and the y-axis represents the speed in m/s. The black dots represent the speed values and the vertical bar shows the MSI at that time. With a window size of 5, the MSI reacts promptly with the change in speed; with a widow size of 30, the MSI reacts weakly. From this experiment, it is clear that we should have a window size of at most 15. A window size of 10 is plausible; however, size of 5 is preferable to 10. Window sizes smaller than five lead to difficulties in obtaining accurate moving window statistics.

In the case where we have several error values in a moving window of size five, it is highly possible for the algorithm to have an erroneous MSI. In such cases, it is preferable to have a bigger window size, even if the reaction of MSI is sacrificed.

In order to solve this dilemma, a window size adjustment mechanism is developed. When a tuple is filtered, this mechanism increases the window size by one to a predefined Maximum Window Size (MWS) and decreases the window size by one to a predefined Initial Window Size (IWS) when a correct tuple is detected. The IWS is set to five and the MWS is set to a value greater than five. This window size adjustment mechanism is implemented in the algorithm as shown in the Algorithm section, and the results of the window size adjustment are presented in the Experimental Verification section.

### Error Correction Methodology

If the speed value calculated for a tuple falls outside the MSI, the tuple has an additional speed value that is larger than the typical speed values. This erroneous tuple is detected and marked as filtered. In order to maintain the moving window statistics, the speed value of the erroneous tuple is restricted to 99.5% of the significant interval. We call this process *calibration*, which actually restricts the speed for maintaining the speed value as large as possible within the MSI at a given time. The purpose of calibration is to avoid possible future underfiltering.

If a tuple is found to have acceleration values larger than *MAX*_*acceleration*_ at a given time, it is clear that the tuple has a position error. The tuple is then detected and filtered. The acceleration error implies that the speed value is erroneous. Instead of calibrating the speed, the erroneous speed value is replaced by *MA*_*speed*_ to maintain the moving window statistics and minimize its effect on the MSI size. For the longitude and latitude, a similar adjustment is made. However, the variance MA (difference) value for the latitude and longitude is used and the directional properties of latitude and longitude must be considered.

**Fig 4:**
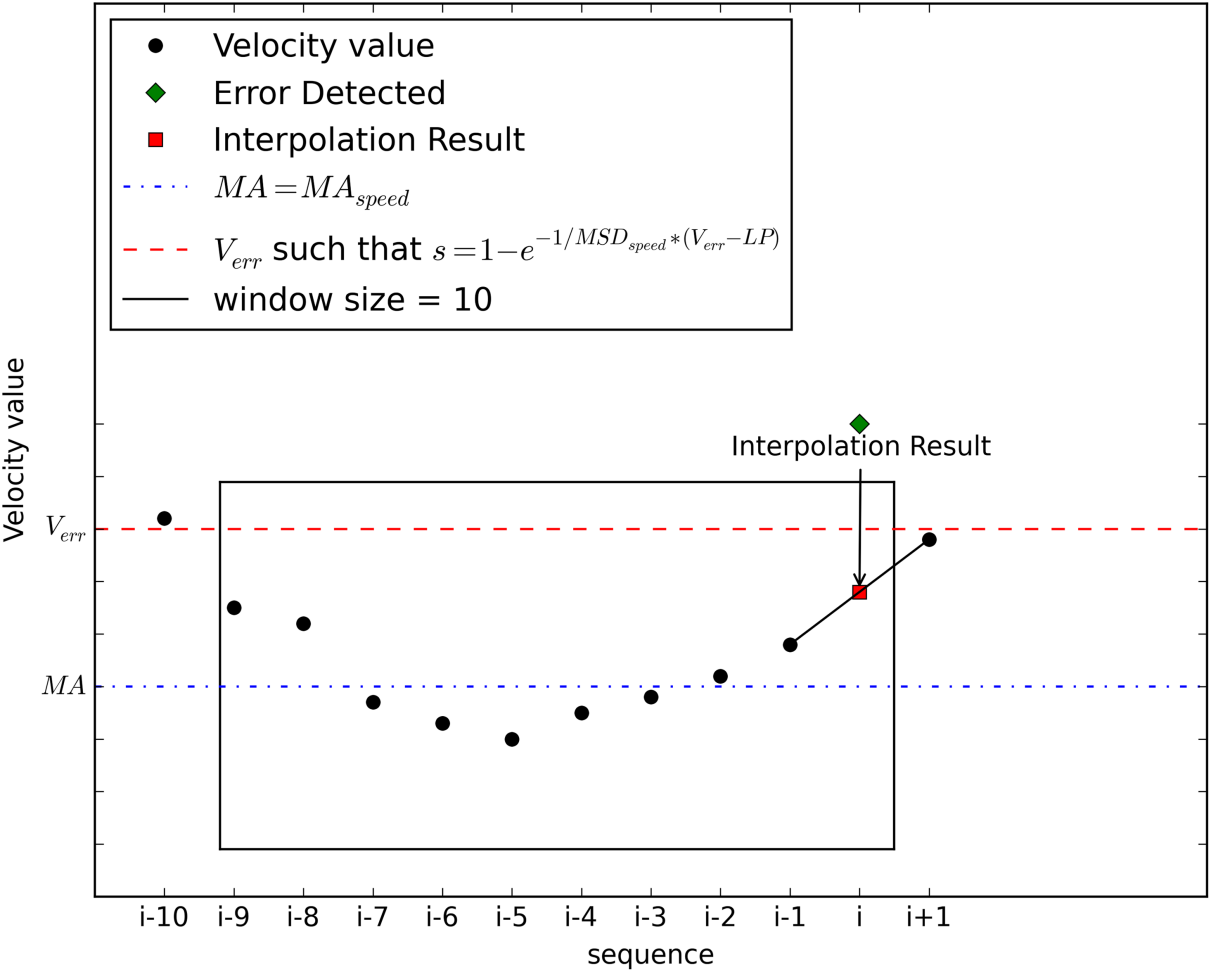
Speed Estimation at the End of the Moving Window.

Once the new speed value *V*_*i* + 1_ is calculated, the speed estimation is processed if *V*_*i*_ is determined as erroneous and is filtered. This implies that *V*_*i*_ has been calibrated. The moving window at time *i* + 1 contains the *n* most recent tuples ending with *P*_*i*_. The speed value at time *i*, *V*_*i*_, is linearly interpolated by *V*_*i* − 1_ and by the newly calculated *V*_*i* + 1_. As discussed earlier, the method for speed estimation is linear interpolation for simpler computation. Fig 4 shows the estimation process with a moving window size of 10. If *V*_*i*_ is determined to be a normal value, no estimation is required.

**Fig 5:**
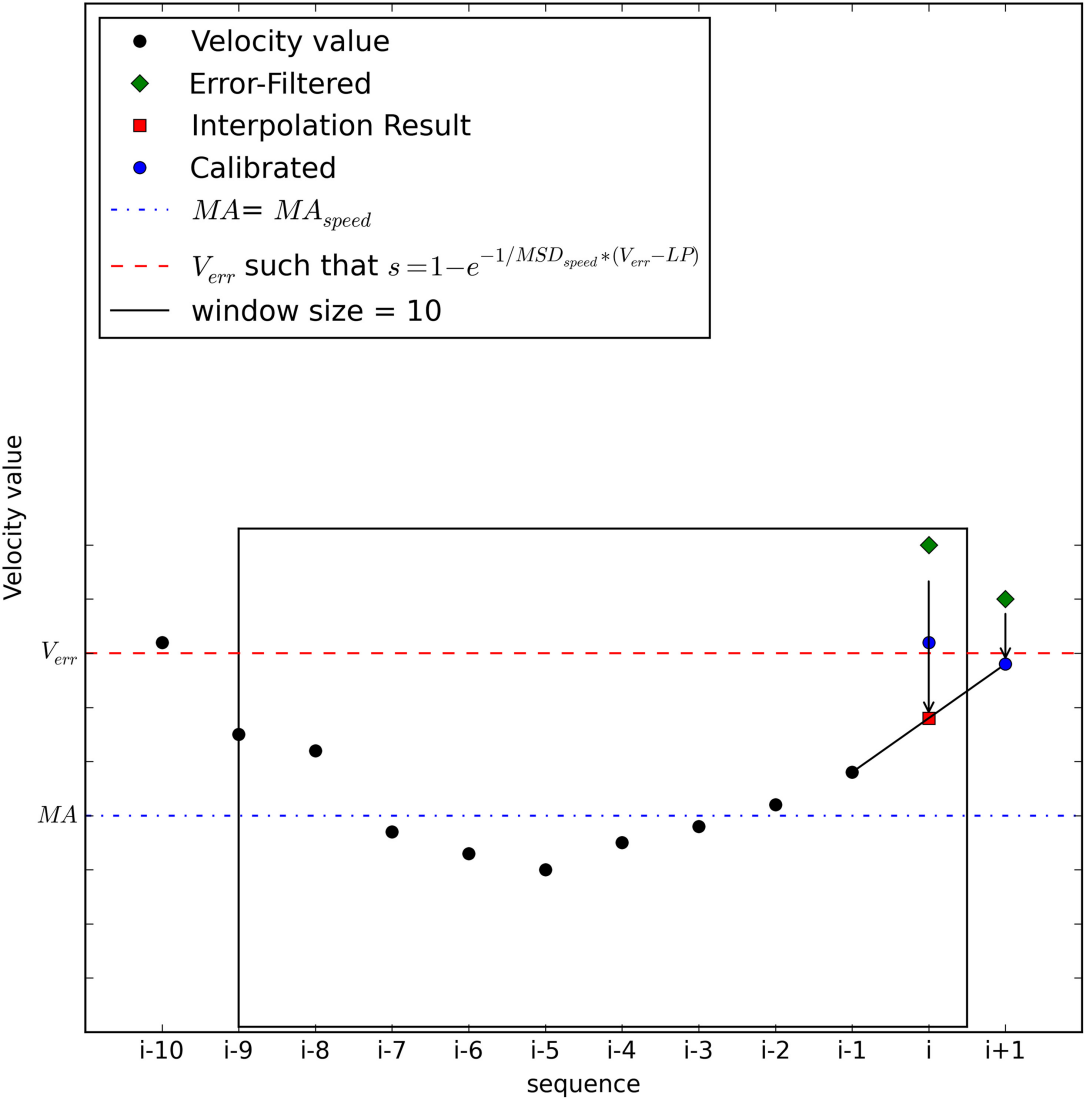
Speed Estimation: A Case of Consecutive Error.

A more complicated case is shown in Fig 5. In the case where the newly calculated *V*_*i* + 1_ is also an erroneous value, *V*_*i* + 1_ must be calibrated first in order to estimate *V*_*i*_. Because a consecutive error is found, speed estimation can be performed only with the best calibrated speed value. If *V*_*i*_ is found to be a normal value, no estimation process is required. Note that there are hidden processes for calibration in Fig 4, but they have been excluded for clearer explanation.

Along with the speed estimation, the location point is also estimated. The process for estimating the longitude and latitude is similar to the speed estimation.

Even when a speed value is found to be erroneous and is filtered, there is always a possibility of overfiltering. If the filtered speed is not an error and is a normal value in comparison with the subsequent speed values, it is considered to have been overfiltered. In such cases, we need to look back and determine whether the filtered speed is within the range of the new MSI. If a past speed value is found to have been overfiltered, the speed value and the longitude and latitude are retrieved. In other words, the original speed, latitude and longitude are restored. We call this a *backtracking* mechanism, the details of which are discussed in the Backtracking subsection.

## Algorithm

Our algorithm accepts consecutive positioning tuples and determines whether the newly arrived tuple is correct or needs to be filtered. It has options for speed estimation, location estimation, etc. We describe the algorithm procedure in this section. The entire algorithm is presented in Algorithm 1.

### Moving Window Construction

Once a new tuple *P*_*i* + 1_ is obtained, the distance between two positions is calculated from the haversine Formulas described in [16] using the positions (latitude and longitude) of *P*_*i* + 1_ and *P*_*i*_. Otherwise, a more complicated method for distance calculation [17] can be used and various alterations can certainly be found. Even the Pythagorean theorem can be used because our algorithm considers relatively small distances only. The distance and time difference between two positions leads to a speed value, *V*_*i* + 1_.

Then, with the *n* most recent tuples, excluding the newly arrived one, the values for the speed moving average (*MA*_*speed*_) and moving standard deviation (*MSD*_*speed*_) are calculated. The same MA value is also calculated for the latitude and longitude variance and referred to as *MA*_*Vlatitude*_ and *MA*_*Vlongitude*_, respectively. In the case of an initial phase of the algorithm where there are less than *n* tuples, constructing a moving window with lesser number of positioning tuples is unavoidable. Part of the algorithm (lines 4 to 17) shows the moving window construction process.

### Moving Significant Interval

After setting the moving window, the speed MSI, latitude variance, and longitude variance can be calculated. The Location Parameter (LP in line 9) is calculated. With the given parameter *s* from the user, an MSI is represented as [**MA**_**speed**_, **V_err_**] where $s=1-e^{-1/{\text{MSD}}_{\text{speed}}*\left(V_{\text{err}}-\text{LP}\right)}$. Two considerations follow: only the positive part of MSI is used, and the minimum value of MSD must be guaranteed (line 10). As discussed, only the positive part of MSI is considered because a decrease in speed could always occur. From the concept of *ET*_*D*_ and MINSPEED, the algorithm calculates the minimum MSD and guarantees the nontrivial size of MSD as indicated in line 5.

### Filtering

Once the calculated speed is larger than the MSI, i.e., an excessive value is found, or the calculated acceleration is abnormal, the tuple is likely to be filtered unless it has a speed value that is below the minimum speed value (line 22). In case possible filtering occurs, the algorithm marks the tuple as filtered, increases the window size by one up to the user-defined maximum size and increases the Number of Consecutive Errors (NCE) (lines 23 to 25). Otherwise, the NCE value is set to zero and the algorithm decreases the current window size by one down to the user-defined IWS (lines 27 to 28).

### Calibration

Excessive speed values that lead to a filtering condition must be restricted within the MSI range. A predefined parameter *S*_99.5_ that represents a 99.5% confidence interval for MSI is used for speed restriction. The speed value *V*_*i* + 1_ is calibrated to *V*_*calib*_ (line 12), where *S*_99.5_ = 1 − *e* ^−1/*MSD*_speed_*(*V*_*calib*_ − *LP*)^, unless it is less than the minimum speed (line 31).

### Restriction by Acceleration

From two consecutive speed values and time, the acceleration value of a tuple can be calculated. Once the acceleration value is larger than the user-defined maximum acceleration, *MAX*_*acceleration*_, the tuple must be filtered as it will have excessive acceleration (lines 33 and 34). Filtering by the acceleration implies that there is a clear error in obtaining the tuple. The acceleration value is replaced by the maximum acceleration value (line 35). Therefore, the speed, latitude, and longitude values of the tuple must be discarded and meaningful values must replace the erroneous ones in order to meaningfully maintain moving window statistics. We choose the MAs values of the speed values as replacements (line 36). The latitude and longitude values are recalculated from the MAs of the variance of differences (lines 37 and 38). We call this mechanism *restriction by maximum acceleration*. If the correct value estimations are activated, those values are replaced by the estimated values. In such cases, lines 35 to 38 of the algorithm are to be optional. Otherwise, the role of acceleration restriction is similar to that of calibration.

### Speed and Location Estimation

In the previous section, we discussed several methods for estimating correct values and simple linear interpolations were chosen because simple computation. The estimations were conducted for cases where the tuple has been marked as *filtered* or *accel_filtered*. Note that a filtered tuple can be restored at the backtracking stage in the following step. However, an *accel_filtered* tuple cannot be restored even when the estimation process is applied (line 40).

### Backtracking

We introduced the backtracking or look back feature for more precise filtering and estimation of erroneous positioning data. We found a tendency for overfiltering especially in the cases where the speed increases. Such overfiltering occurs because the increase in MSI does not correspond to the increase in speed with time. Instead, a tuple with increased speed is filtered even when it corresponds to correct positioning data. In order to avoid overfiltering of correct positioning data, we introduced backtracking. At a given time and for a given MSI, the algorithm looks back on *V*_*i*_ in order to check whether it is filtered. If it is inside the range of MSI, the tuple with *V*_*i*_ is recovered, the consecutive moving window contains the retrieved *V*_*i*_, and the backtracking mechanism eventually causes the moving window statistics calculation to reflect the retrieved *V*_*i*_.

### Window Size Adjustment

In erroneous situations, the window size can be increased or decreased. User-defined parameters, such as IWS and MWS are required to be known. If the algorithm finds an erroneous tuple, it increases the window size by one (lines 25) up to MWS. If the algorithm finds a normal tuple, or a previously filtered tuple is found to be retrieved, the algorithm decreases the window size by one down to IWS (lines 28 and 46) and NCE is set to zero (line 45). We recommend setting IWS to five. MWS can be dependent upon the memory capacity of the devices that execute the algorithm under the condition that *IWS* ≤ *MWS*.

### Input Requirement

The algorithm requires several parameters to be predefined by the users, as shown in the initial part of algorithm 1. A sequence of tuples is, naturally, a mandatory input, in addition to IWS and MWS for the boundaries of the window size adjustment. Furthermore, the user-defined SL *s*, minimum human ambulation speed *MINSPEED*, and the environmental error tolerance of distance *ET*_*D*_ are required.

**Algorithm 1**: Position Error Detection and Estimation

**Require**: series of *P*_*i*_, *t* > *i* > 0 if the series exists or *P*_0_

**Require**: *P*_0_ // At least one initial tuple is required

**Require**: IWS, MWS, window size *n* = *IWS* // *IWS* ≤ *MWS*

**Require**: user SL *s*, error tolerance of distance *ET*_*D*_, minimum speed *MINSPEED*

1: i = 0

2: NCE = 0

3: **repeat**

4:  **Get** *P*_*i* + 1_ // Acquisition of new tuple, if exist

5:  **Set** *MIN*_*speed*_ = (*ET*_*D*_/(*t*_*i* + 1_ − *t*_*i*_)>*MINSPEED*) ? *ET*_*D*_/(*t*_*i* + 1_ − *t*_*i*_):*MINSPEED*

6:  **Construct** *MA*_*speed*_(*n*) with {*P*_*x*_: *max*(*i* − *n* + 1, 0) ≤ *x* ≤ *i*}

7:  **Construct** *MSD*_*speed*_(*n*) with {*P*_*x*_: *max*(*i* − *n* + 1, 0) ≤ *x* ≤ *i*}

8:  **Set** *MA*_*speed*_ = *MA*_*speed*_(*n*)

9:  **Set** *LP* = *MA*_*speed*_ − *MSD*_*speed*_

10:  **Set** *MSD*_*speed*_ = (*MSD*_*speed*_(*n*)>*MIN*_*speed*_)? *MSD_speed_*(*n*): *MIN_speed_* // Allow minimum room for moving significant interval

11:  **Compute** *V*_*err*_ such that *s* = 1 − *e* ^−1/*MSD*_speed_*^(*V*_*err*_ − *LP*) // Compute Maximum possible velocity in user SL s

12:  **Compute** *V*_*calib*_ such that 0.995 = 1 − *e* ^−1/*MSD*_speed_*(*V*_*calib*_ − *LP*)^ // Compute velocity for checking whether *V*_*i* + 1_ is to be calibrated

13:  **Set** *Vlat*_*i* + 1_ = ∥*lat*_*i* + 1_, *lat*_*i*_∥/(*t*_*i* + 1_ − *t*_*i*_)

14:  **Construct** *MA*_*Vlatitude*_(*n*) with {*P*_*x*_: *max*(*i* − *n* + 1, 0) ≤ *x* ≤ *i*}

15:  **Set** *MA*_*Vlatitude*_ = *MA*_*Vlatitude*_(*n*)

16:  **Set** *Vlon*_*i* + 1_ = ∥*lon*_*i* + 1_, *lon*_*i*_∥/(*t*_*i* + 1_ − *t*_*i*_)

17:  **Construct** *MA*_*Vlongitude*_(*n*) with {*P*_*x*_: *max*(*i* − *n* + 1, 0) ≤ *x* ≤ *i*}

18:  **Set** *MA*_*Vlongitude*_ = *MA*_*Vlongitude*_(*n*)

19:  **Set** *lat*_*i* + 1, *original*_ = *lat*_*i* + 1_

20:  **Set** *lon*_*i* + 1, *original*_ = *lon*_*i* + 1_

21:  **Set** *V*_*i* + 1, *original*_ = *V*_*i* + 1_ = *dist*(*P*_*i* + 1_, *P*_*i*_)/(*t*_*i* + 1_ − *t*_*i*_) // dist(): distance between two points

22:  **if** ((*V*_*i* + 1_ > *V*_*err*_) OR (*a*_*i* + 1_ ≥ *MAX_acceleration_*)) AND (V _i + 1_ > *MIN_speed_*) **then**

23:   **Mark** *P*_*i* + 1_ as filtered. // Filtering

24:   NCE++

25:   *n* = (*n* + 1 > *MWS*)?*MWS*: *n*++ // Window Size Adjustment-Increase

26:  **else**

27:   Set NCE = 0

28:   *n* = (*n* − 1 < *IWS*)?*IWS*: *n* − − // Window Size Adjustment-Decrease

29:  **end if**

30:  **if** (*V*_*i* + 1_ ≥ *V*_*calib*_) AND (*V*_*i* + 1_ > *MIN*_*speed*_) **then**

31:   **Set** *V*_*i* + 1_ = *V*_*calib*_ // Calibration of Speed

32:  **end if**

33:  **if** *a*_*i* + 1_ ≥ *MAX*_*acceleration*_ **then**

34:   **Mark** *P*_*i* + 1_ as accel_filtered // Restriction by the Maximum Acceleration

35:   **Set** *a*_*i* + 1_ = *MAX*_*acceleration*_

36:   **Set** *V*_*i* + 1_ = *MA*_*speed*_

37:   **Set** *lat*_*i* + 1, *corrected*_ = *lat*_*i*_ + *sign*(*lat*_*i* + 1_ − *lat*_*i*_) × *MA*_*Vlatitude*_ × (*t*_*i* + 1_ − *t*_*i* − 1_)

38:   **Set** *lon*_*i* + 1, *corrected*_ = *lon*_*i*_ + *sign*(*lon*_*i* + 1_ − *lon*_*i*_) × *MA*_*Vlongitude*_ × (*t*_*i* + 1_ − *t*_*i* − 1_)

39:  **end if**

40:  **if** (*V*_*i*, *original*_ ≤ *V*_*err*_) and (*P*_*i*_ marked as filtered) and !(*P*_*i*_ marked as accel_filtered) **then**

41:   **Mark** *P*_*i*_ as retrieved

42:   *V*_*i*_ = *V*_*i*, *original*_

43:   *lat*_*i*_ = *lat*_*i*, *original*_

44:   *lon*_*i*_ = *lon*_*i*, *original*_ // Backtracking: Look back one step and Restore with original values

45:   **Set** NCE = 0

46:   *n* = (*n* − 1 < *IWS*)?*IWS*: *n* − − // Window Size Adjustment-Decrease

47:  **end if**

48:  **if** (*P*_*i*_ marked as filtered or accel_filtered) **then**

49:   **Set** *V*_*i*_ = (*V*_*i* + 1_ − *V*_*i* − 1_) × (*t*_*i*_ − *t*_*i* − 1_)/(*t*_*i* + 1_ − *t*_*i* − 1_) + V i-1 // Estimation of speed

50: **Set** *lat*_*i*_ = (*lat*_*i* + 1_ − *lat*_*i* − 1_) × (*t*_*i*_ − *t*_*i* − 1_)/(*t*_*i* + 1_ − *t*_*i* − 1_) + *lat*_*i* − 1_

51:   **Set** *lon*_*i*_ = (*lon*_*i* + 1_ − *lon*_*i* − 1_) × (*t*_*i*_ − *t*_*i* − 1_)/(*t*_*i* + 1_ − *t*_*i* − 1_) + lon i-1 // Estimation of Position

52:   **Mark** *P*_*i*_ as interpolated

53:  **end if**

54:  **Set** i = i + 1

55: **until** Exist no more input of positioning tuple

## Experimental Verification

In this section, experiment results with the actual collected positioning data are presented. Based on the steps of the algorithm, corresponding results with graphs and maps are provided in this section.

### Effect of *ET*_*D*_ and SL

**Fig 6:**
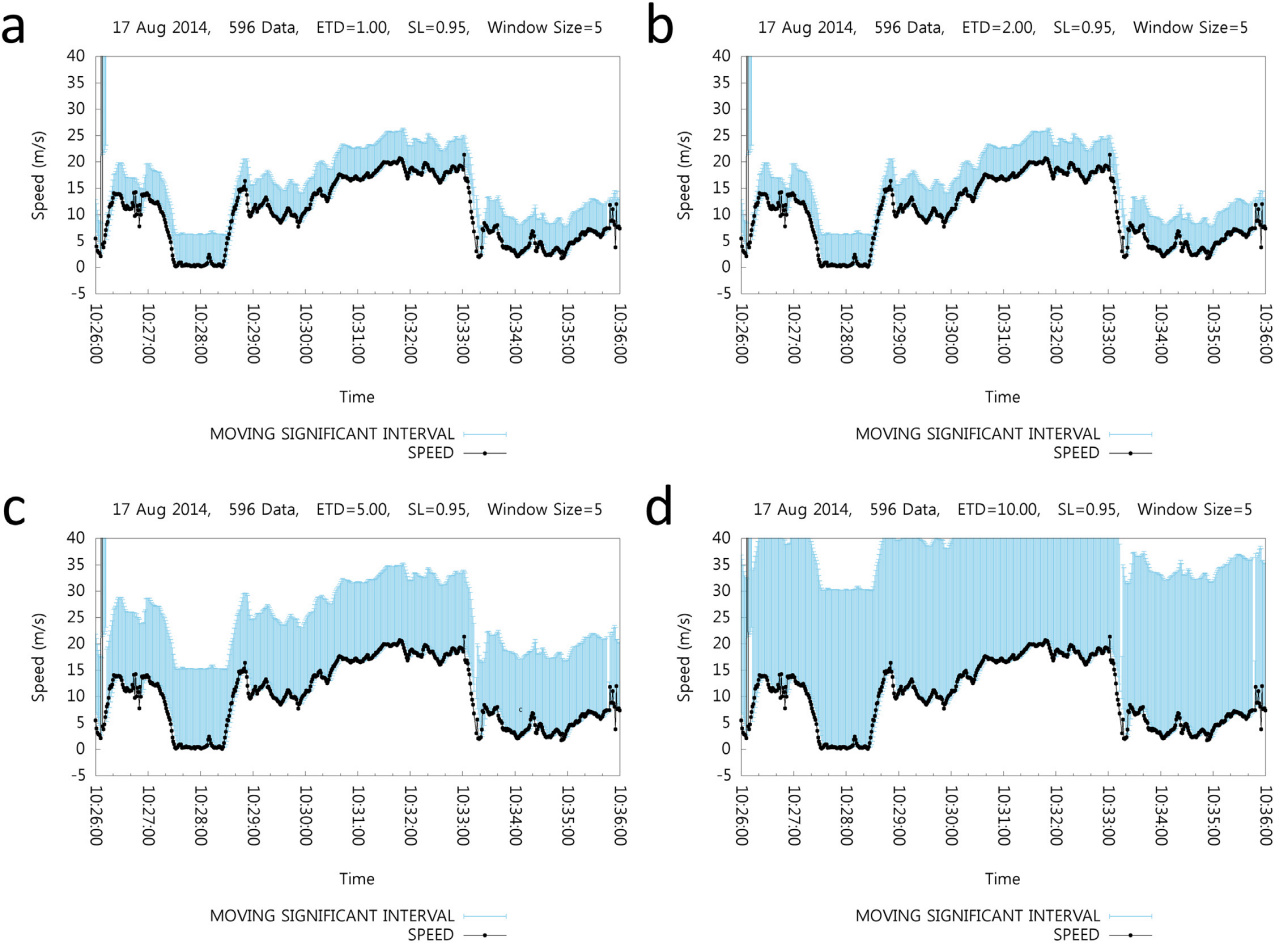
Effect of Distance Error Tolerance *ET*_*d*_.

The SL effects, or significant level, and the error tolerance of distance need to be identified first. By varying the *ET*_*D*_ and *SL* values, the corresponding MSI length must be observed in order to determine appropriate values for *ET*_*D*_ and *SL*. Fig 6 shows the MSI length for various *ET*_*D*_ values, i.e., 1.0, 2.0, 5.0, and 10.0. Note that *ET*_*D*_ is one of the user-defined parameters that are a part of the environmental parameters, which represents one of the parametric values for positioning system errors. On the top of each subfigure, the date of the positioning data collection, data count, *ET*_*D*_ value, value of the significant level (*SL*), and window size are shown. The x-axis represents the time of day, and the y-axis represents the speed values. The *ET*_*D*_ values in this experiment are selected from Table 4. Each subfigure shows the MSI according to the given *ET*_*D*_ value. In each subfigure, the black dot denotes the speed value at that time, and the vertical bar shows the MSI range at that given time. Fig 6a, 6b, 6c and 6d correspond to *ET*_*D*_ value set as 1.0, 2.0, 5.0, and 10.0, respectively. Larger *ET*_*D*_ values naturally lead to a larger MSI.

For the notable cases of *ET*_*D*_ at 1.0 and 2.0, the MSI length shown in Fig 6a and 6b are the same. This is because of the effect of *MINSPEED*, which is set at 2.0*m*/*s*; line 5 of algorithm 1 sets *MIN*_*speed*_ to 2.0 when the *ET*_*D*_ effect is less than *MINSPEED* of 2.0. Therefore, the minimum MSI length is approximately 5.99*m*/*s* whenever *MSD*_*speed*_ is equal to or less than 2.0 as *MINSPEED*.

**Fig 7:**
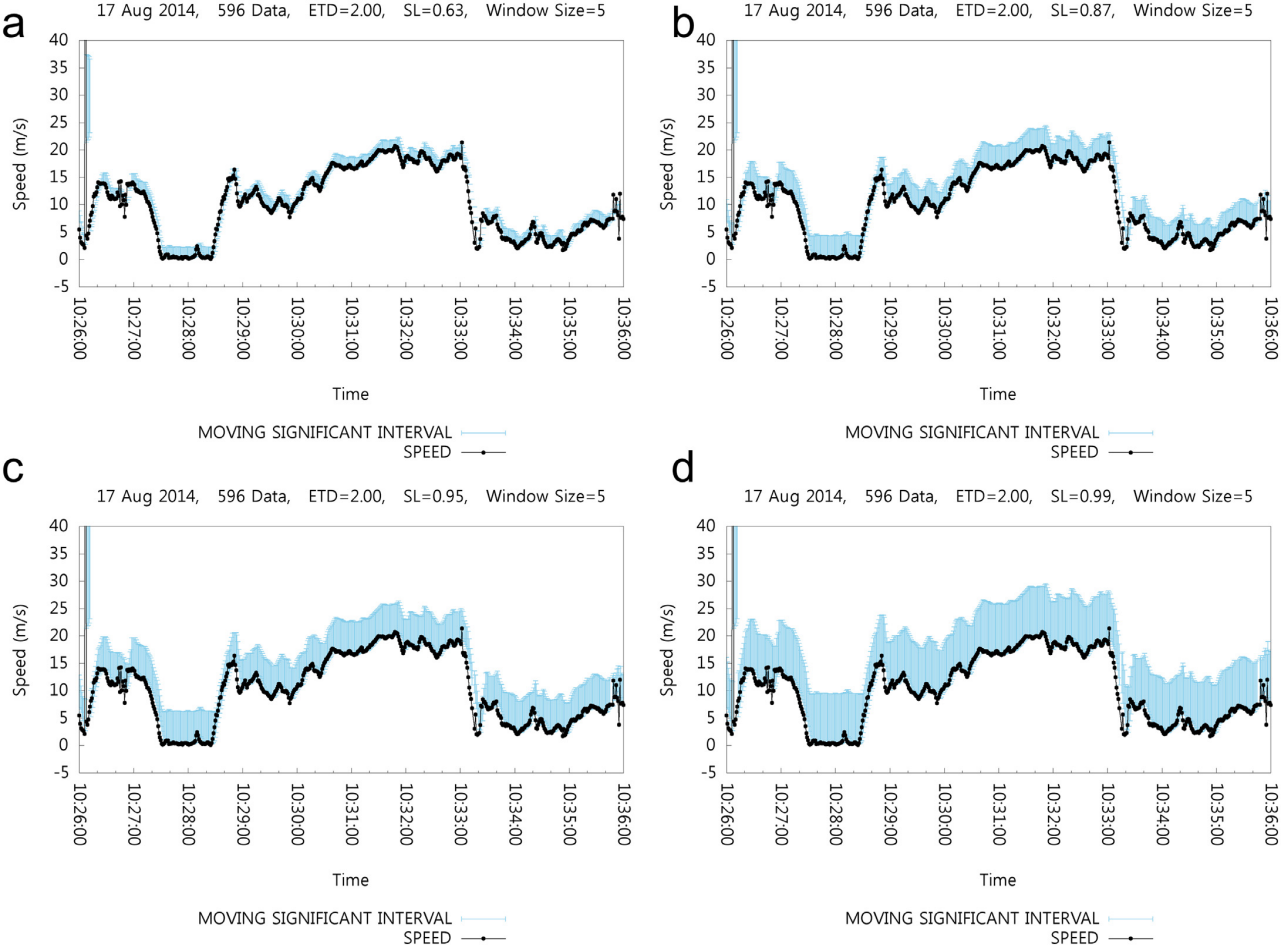
Effect of the Significant Level.

Fig 7 shows the effect of SL. Significant levels for the significant intervals are set as 0.63, 0.87, 0.95 and 0.99 and the corresponding MSI values are shown in each subgraph. SL can be defined by the user as the estimated rate of positioning error. For example, the rate of error in the case of outdoor cellular base station positioning systems can be 36.9%, as indicated in Table 1. Naturally, the error rate can be different in other areas or with other positioning devices. However, this is the only estimation. In such situations, the SL value can be set at 0.63, which means that 63% of the positioning data are correct. Through the SL value, the users of this algorithm can control the MSI range.

Again, each subfigure shows the MSI range with the same positioning data set. The same positioning data set and legends are used in Fig 6. For each case, Fig 7a shows the SI as 0.63; Fig 7b, 7c, and 7d show SI as 0.87, 0.95, and 0.99, respectively.

From the basic test of *ET*_*D*_ and *SL*, it is plausible to set *ET*_*D*_ as low as possible (but to an appropriate value) and control the size of the MSI by SL. For the remainder of the experiments, the aforementioned parameters are typically set as *ET*_*D*_ = 2, *SL* = 0.95, and window size to five, unless specified otherwise.

### Detection of Erroneous Speed

**Fig 8:**
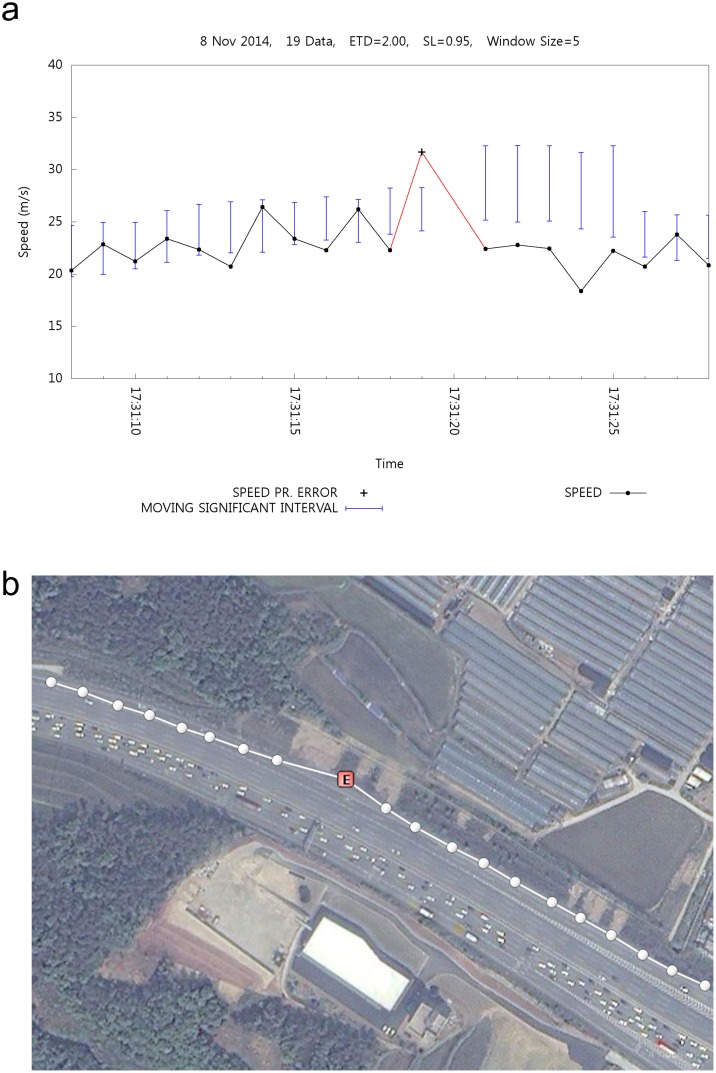
Speed Error Detection.

Fig 8 shows the effect of erroneous speed detection and filtering. Fig 8a shows the time-speed graph with speed values and the corresponding MSI. The x-axis represents the time of day and the y-axis represents the speed values. The black dots (⋅) represent normal speed values, whereas (+) represents the filtered erroneous speed values. The vertical bar shows the MSI length above the MA at a given time. Once a calculated speed exceeds the MSI at a given time, a speed error is detected, and such positioning data are also marked as erroneous. In Fig 8b, the white circles show the position with normal speeds, whereas the red [E] marks show the position with erroneous speeds. As shown in Fig 8a, a speed error is detected at time 17:31:19. Fig 8b shows the corresponding erroneous position on the map. A speed error is detected because it is outside the MSI.

### Calibration of Erroneous Speed Value

**Fig 9:**
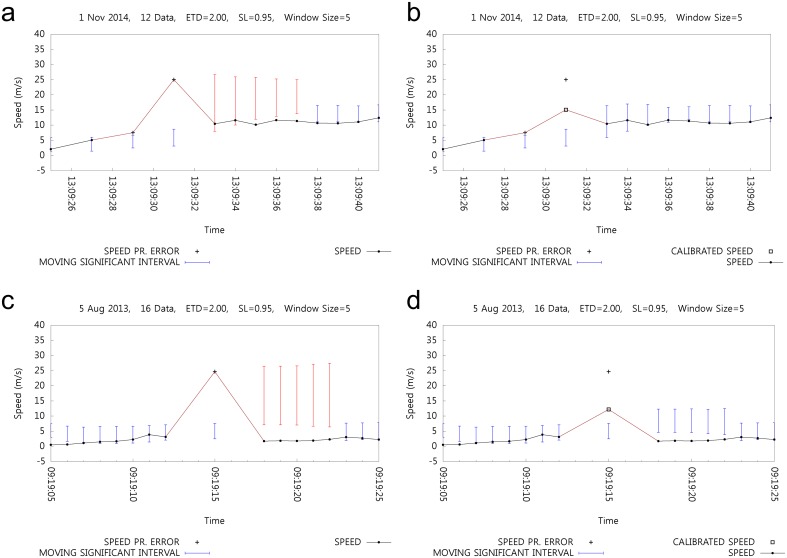
Calibration of Erroneous Speeds.

Fig 9 shows two cases of speed error calibration. Fig 9a and 9c show the detection of speed errors without speed error calibration. Fig 9b shows the effect of speed error calibration denoted as □ that corresponds to Fig 9a. Without the calibration process, the subsequent MSIs are affected with erroneous speeds up to the size of the window. The same effect can be seen in Fig 9c, whereas Fig 9d shows the effect of calibration on the MSI. Fig 9a shows an erroneous speed at 13:09:31, and the subsequent MSI can cause underfiltering for the following speed errors. As mentioned in the Calibration subsection, erroneous speeds can cause subsequent MSIs to be erroneous and result in underfiltering. Calibration of erroneous speeds reduces the effect on MSI and hence reduces underfiltering, as shown in Fig 9b and 9d.

### Restriction by Acceleration Error

**Fig 10:**
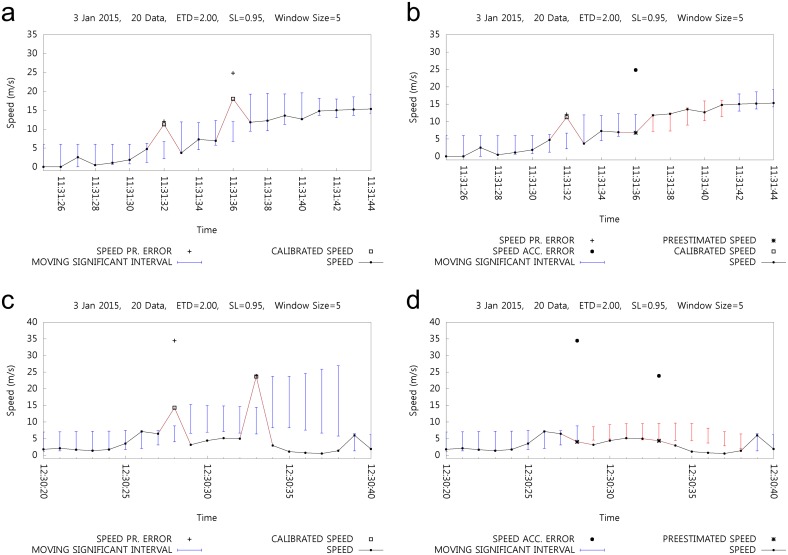
Effect of Acceleration Error.

Speed errors detected by unreal acceleration values need to be managed and restricted to normal speed values. Fig 10 shows the case of speed value restriction due to acceleration with the criterion of the maximum acceleration, *MAX*_*acceleration*_ = 10.8*m*/*s* ^2^. The same calibration mechanism can naturally be applied in this situation, whereas a different treatment is required because the error of acceleration represents no overfiltering, i.e., the error of acceleration is obvious. Fig 10a shows the case of calibration when the acceleration error is treated as a speed error. Two errors occur at 11:31:32 and 11:31:36; the first error is not an acceleration error but the second error is an acceleration error. Fig 10b shows the application of another treatment to the acceleration error. In the graph, the • symbol represents the detected acceleration error. Because algorithm 1 replaces the erroneous speed value with *MA*_*speed*_, the subsequent MSIs have a minimized effect on the speed error by acceleration. Fig 10c shows another case of acceleration error with calibration, and correspondingly Fig 10d clearly shows the restriction effect of the acceleration error on the MSI. At 12:30:28 and 12:30:33, two acceleration errors are restricted and consequently, the following MSIs are stabilized.

### Speed Estimation

**Fig 11:**
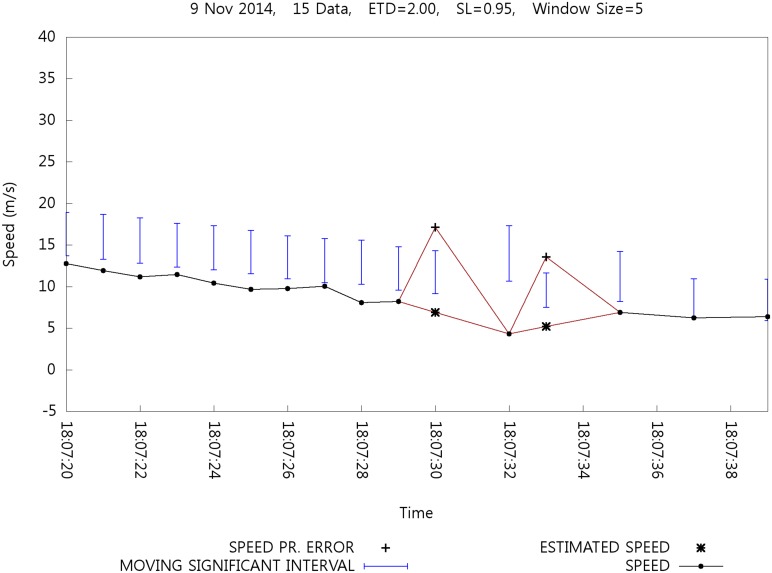
Effect of Speed Estimation.

The result of estimation is shown with the effect of erroneous speed detection. Fig 11 shows the outcome of speed estimation. The symbol ✳ in the graph shows the estimated speed at a given time. In this case, speed estimation occurs at 18:07:30 and 18:07:33. Note that the speed estimation cannot be performed at the time of error detection. At time *i* + 1, it is determined whether position data *P_i_* are erroneous, and then speed estimation can be performed because position data *P*_*i* + 1_ and *P*_*i* − 1_ must be used to estimate speed *P_i_*. Therefore, calibration is still effective for speed estimation. At 18:07:32, the speed estimation does not affect the size of the MSI; instead, the speed estimation at 18:07:30 is completed, and the estimated speed at 18:07:30 cannot be included for calculating the MSI at 18:07:32. A similar phenomenon can be observed at 18:07:33. However, the estimated speed values eventually affect the size of successive MSIs.

### Location Estimation

**Fig 12:**
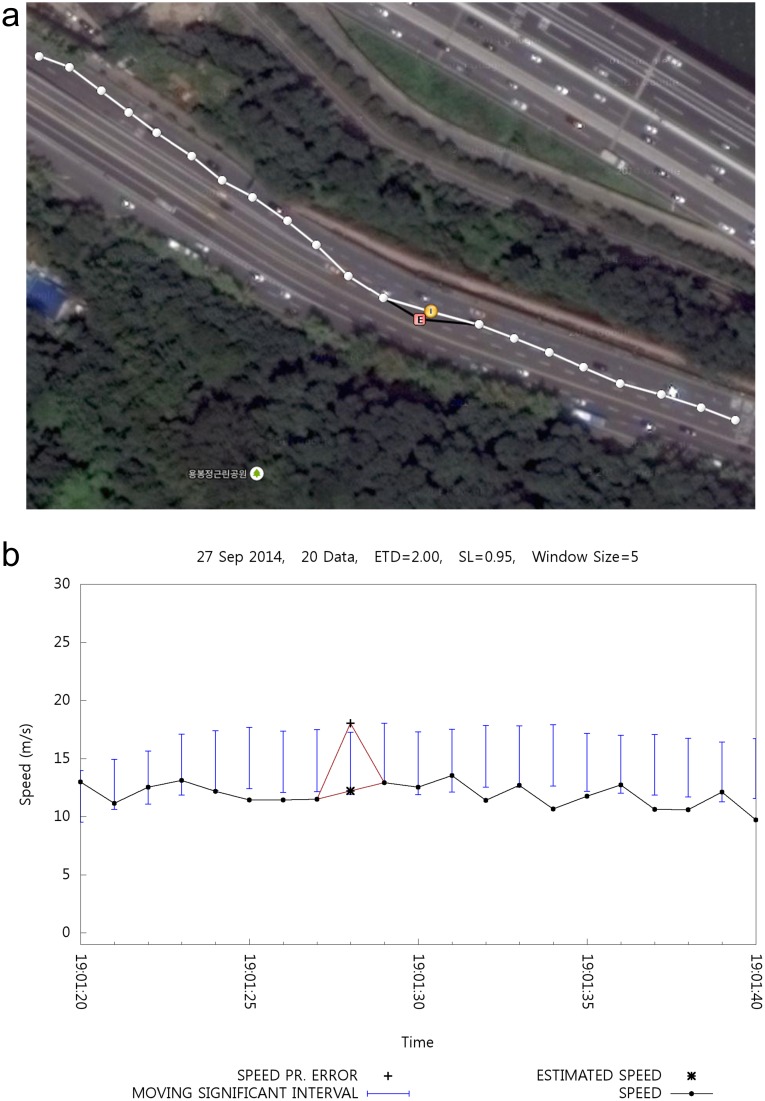
Effect of Location Estimation.

Similar to speed estimation, it is possible to estimate the location. Fig 12 shows the result of position data estimation. Fig 12a is a map that contains the position of the plotted movement. Such movement has been recorded from right to left. The big circle represents the estimated position, whereas mark E within the red box shows the corresponding erroneous position. Fig 12b shows the time-speed graph that corresponds to Fig 12a.

### Backtracking

Calibration, restriction by acceleration, and speed estimation consider the underfiltering of erroneous speed values, whereas the backtracking mechanism is prepared to cope with the overfiltering of normal speed values. Backtracking can be combined with either speed calibration or estimation.

**Fig 13:**
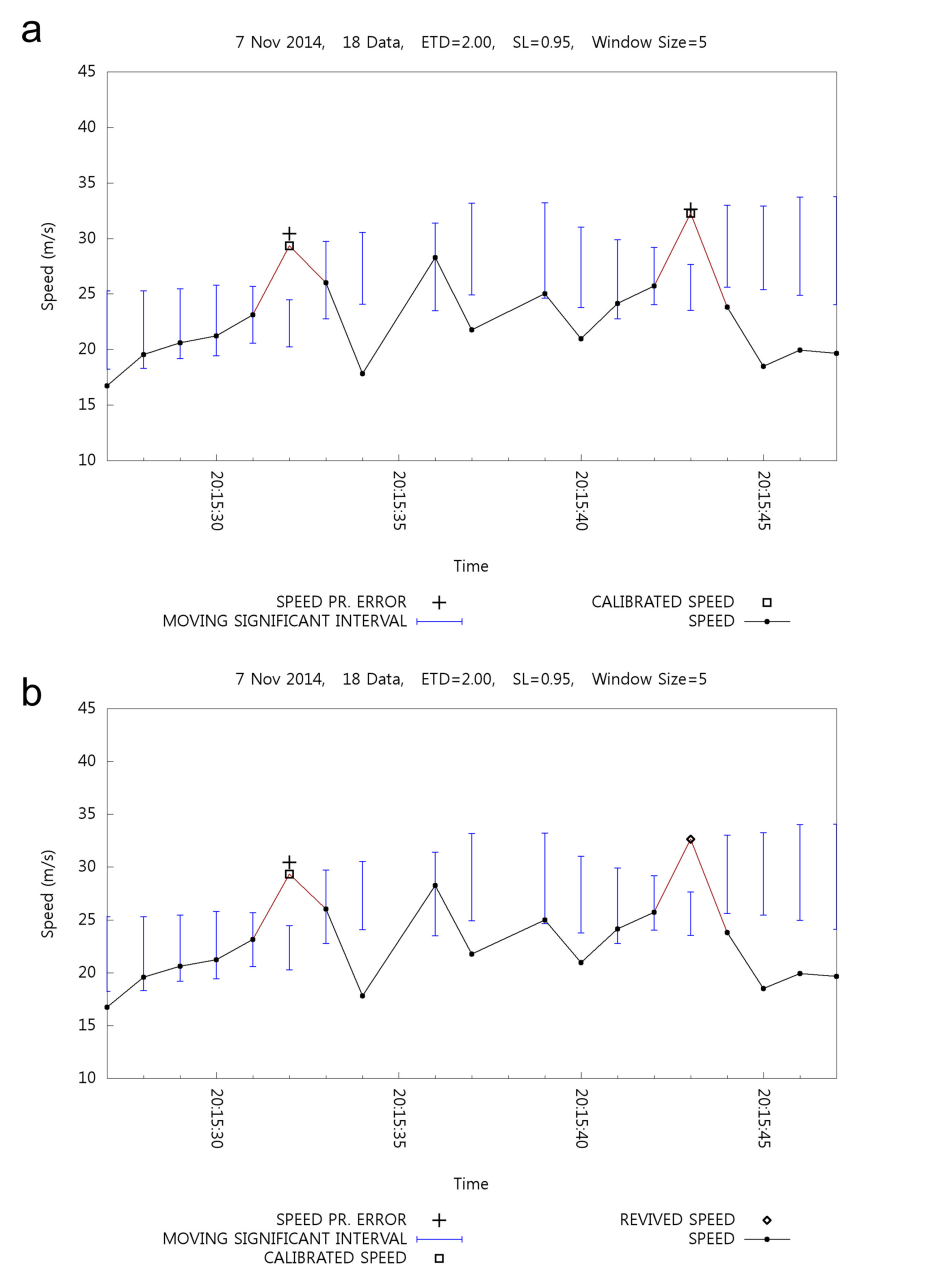
Effect of Backtracking with Speed Calibration.

Fig 13 shows the effect of calibration and backtracking. Fig 13a shows calibration without backtracking where two errors occur at 20:15:32 and 20:15:43, and are then calibrated. Fig 13b shows the result of the backtracking mechanism where one of the speeds is revived at 20:15:43, denoted by ⋄ symbol.

Table 5 lists the related statistics in order to observe the details of the backtracking effect. Backtracking revives 132 positioning data out of 155 erroneous positioning data. Backtracking causes slight increments in the average speed and decrements of filtering ratio. Fig 13 shows the results of the partial data listed in Table 5.

**Table 5:** Statistics for the effect of backtracking in combination with Speed Estimation. (Window size = 5, *ET*_*D*_ = 2.0, SL = 0.95.)

**Statistics values**	**Calibration Only**	**Calibration with Backtracking**
Total Data	11,891	
Raw Average Speed (m/s)	25.9875	
Raw Maximum Speed (m/s)	604.303	
Number of Filtered Data	155	155
Number of Recovered Data	N/A	132
Filtered Average Speed (m/s)	25.7203	25.7207
Filtered Maximum Speed (m/s)	595.491	595.491
Filtering Ratio (%)	1.30351	0.193424

**Fig 14:**
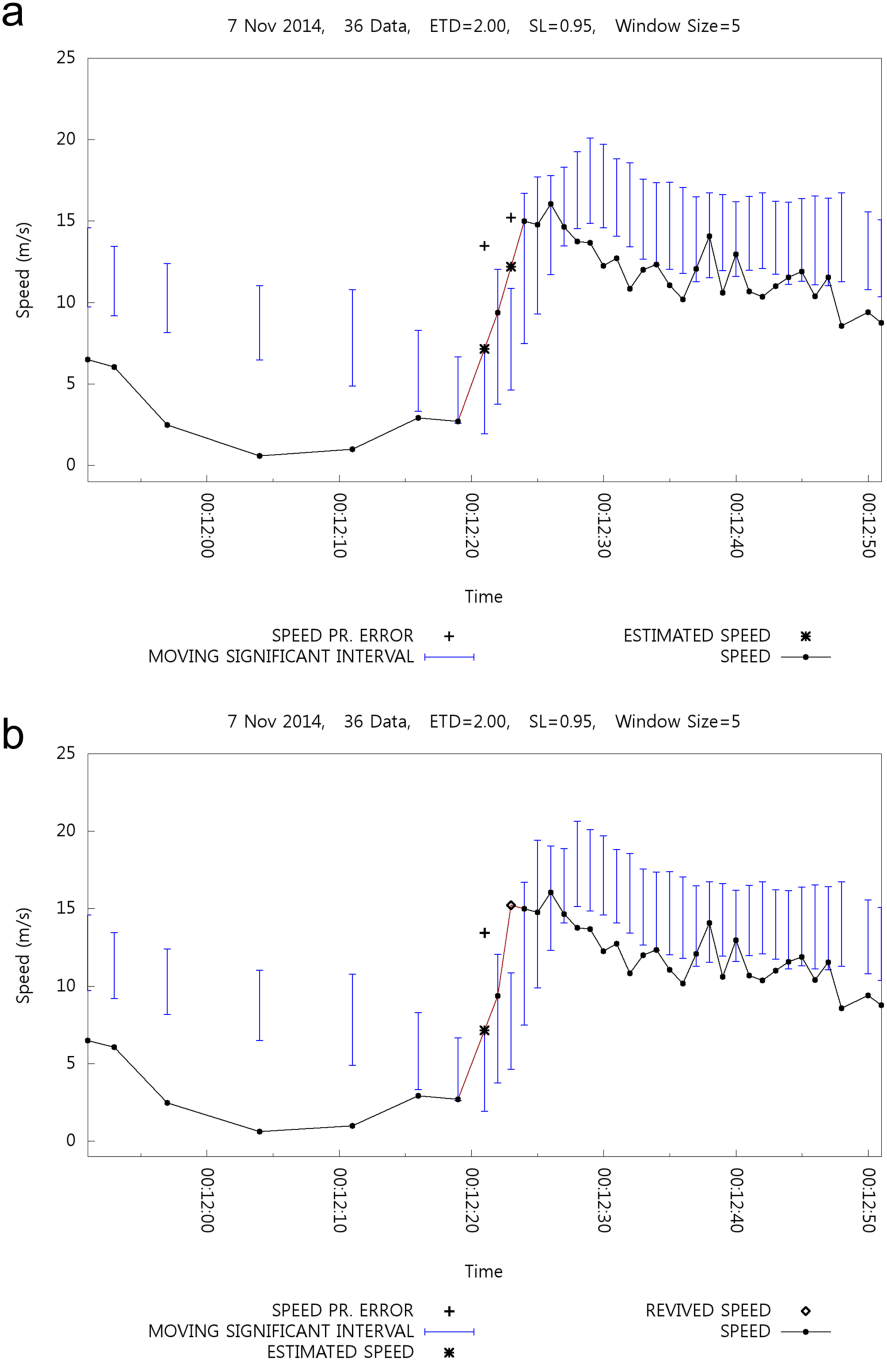
Effect of Backtracking with Speed Estimation.

Fig 14 shows the effects of backtracking in combination with speed estimation. Fig 14a shows the case without backtracking. Errors occur at 00:12:21 and 00:12:23, and they are then estimated. Fig 14b shows the case that includes backtracking. The speed value at 00:12:23 is revived and the estimated speed value is discarded, whereas the speed value at 00:12:21 cannot be revived.

This figure shows overfiltering in the case of rapid speed increment. Such a rapid increment of speed can cause overfiltering, and the backtracking mechanism revives the overfiltered positioning data.

Table 6 lists the related statistics for the data from an entire day. Fig 14 shows only the portion of data listed in Table 6. Similar to the case of calibration in combination with backtracking, the filtering ratio decreases here. The average of the estimated speed to be revived is naturally smaller than that of the revived speed. The average gap between the estimated and revived speed is approximately 4 m/s and the maximum difference (gap) is more than 21 m/s. Considering that speed and location estimation are preferred to only calibration, it is better to combine backtracking with the estimation mechanism. Note that Tables 5 and 6 list the statistics of different data sets, although Figs 13 and 14 show the result for the same day. Two different positioning devices were used by two different researchers.

**Table 6:** Statistics for the effects of backtracking in combination with Speed Estimation. (Window size = 5, *ET*_*D*_ = 2.0, SL = 0.95.)

**Statistics values**	**Estimation Only**	**Estimation with Backtracking**
Total Data	13,406	
Raw Average Speed (m/s)	23.2345	
Raw Maximum Speed (m/s)	621.224	
Number of Filtered Data	145	136
Number of Retrieved Data	N/A	80
Filtered Average Speed (m/s)	21.7735	21.8008
Filtered Maximum Speed (m/s)	65.8308	65.8308
Filtering Ratio (%)	1.08161	0.417723
Average of Estimated Speed before revival (m/s)	9.305648	
Average of Retrieved Speed (m/s)	13.30535	
Average of gap (m/s)	3.999706	
Maximum of gap (m/s)	21.08463	

### Window Size Adjustment

MSI fluctuates immediately after the detection of speed error, but this is unavoidable. In order to cope with this fluctuation, increasing the window size for some time could be a solution. Moreover, window size increment can also be useful for managing continuous errors.

**Fig 15:**
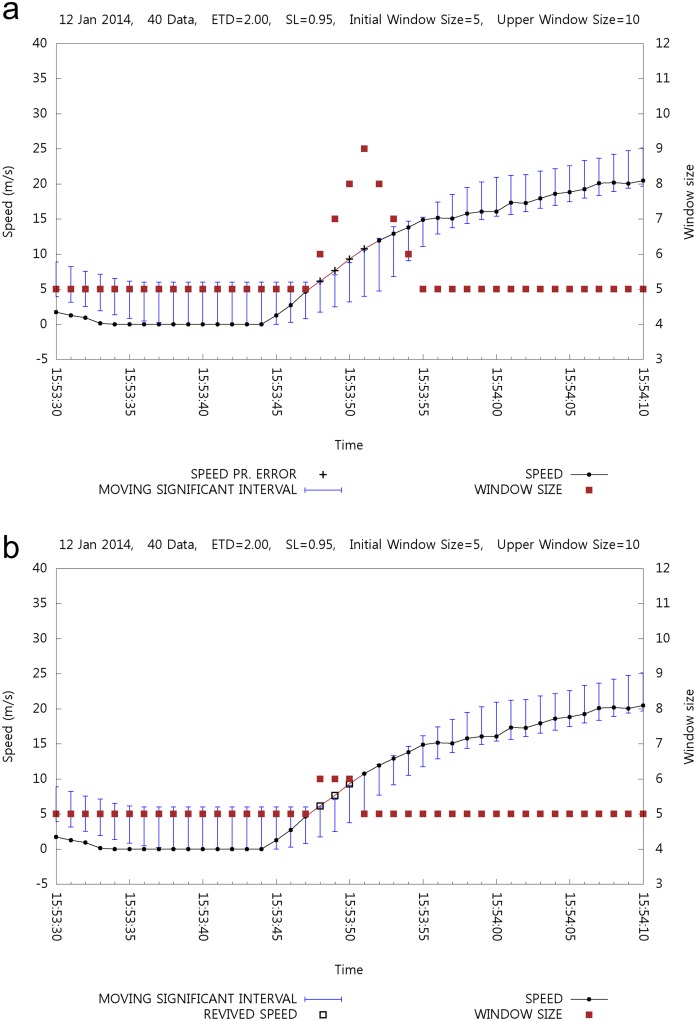
Window Size Adjustment.

Experiments with window size adjustment are made. Fig 15 shows the case of a window size adjustment with and without estimation and backtracking. The right y-axis represents the window size and ■ shows such size in the graph at a given time. Note that the window size at a specific time affects the size of the successor MSI. Fig 15a shows the window size adjustment without estimation and backtracking. At 15:53:48, the first error is detected and four consecutive errors are detected until 15:53:51. Then the window size increments. The window size becomes nine at 15:53:51, and the window size decreases with the normal speed values. Fig 15b shows the case of windows size adjustment with estimation and backtracking. At time 15:53:48, the first speed error is detected and the window size increases to six. Consequently, at 15:53:49, speed error detection increases the window size to seven, but the revival by backtracking decreases the window size to six. However, backtracking revives the successive speed values, and the window size is decreased to five.

Table 7 shows a comparison of the statistics between fixed and varying window sizes for a positioning data set. For a varying window size, IWS is five and MWS is ten. As expected, window size adjustment avoids the MSI fluctuation so that more suspicious speed errors are detected. In this case, less speed values are revived and the average speed becomes lower than in the case of a fixed window size.

**Table 7:** Statistics for the effect of Window Size Adjustment.

**Statistics values**	**Fixed Window Size**	**Varying Window Size**
Total Data	15,090	
Maximum Speed (m/s)	35.9159	
Average Speed (m/s)	15.1134	15.1074
Number of Filtered Data	199	203
Maximum Number of Continuous Filtered Data	3	4
Number of Retrieved Data	41	28
Filtering Ratio (%)	1.04705	1.15971

**Fig 16:**
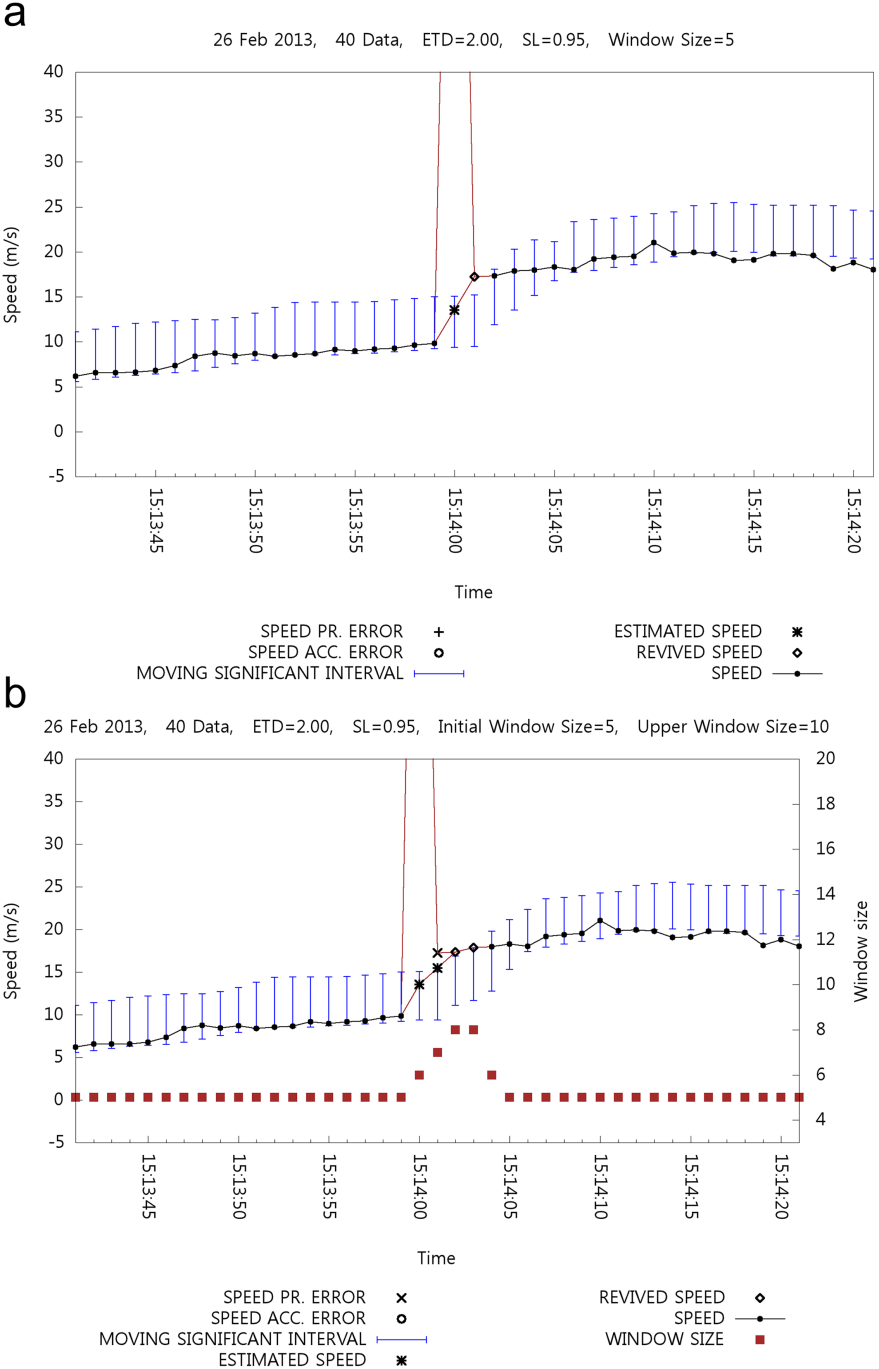
Case1: Effect of Window Size Adjustment.

For example, Fig 16 shows the graphs of a typical part of the data listed in Table 7, where a typical speed error is revived with a fixed size window whereas the speed error is filtered with a variable size window. Fig 16a shows the case of a fixed window size of five, whereas Fig 16b shows the case of varying window size. In Fig 16a, speed error by acceleration is detected at 15:14:00, and then the speed restriction is applied. The speed error at 15:14:01 is eventually revived by backtracking. However, in Fig 16b, the speed error at 15:14:01 is not revived. In addition, the errors at 15:14:02 and 15:14:03 are detected and retrieved later.

**Fig 17:**
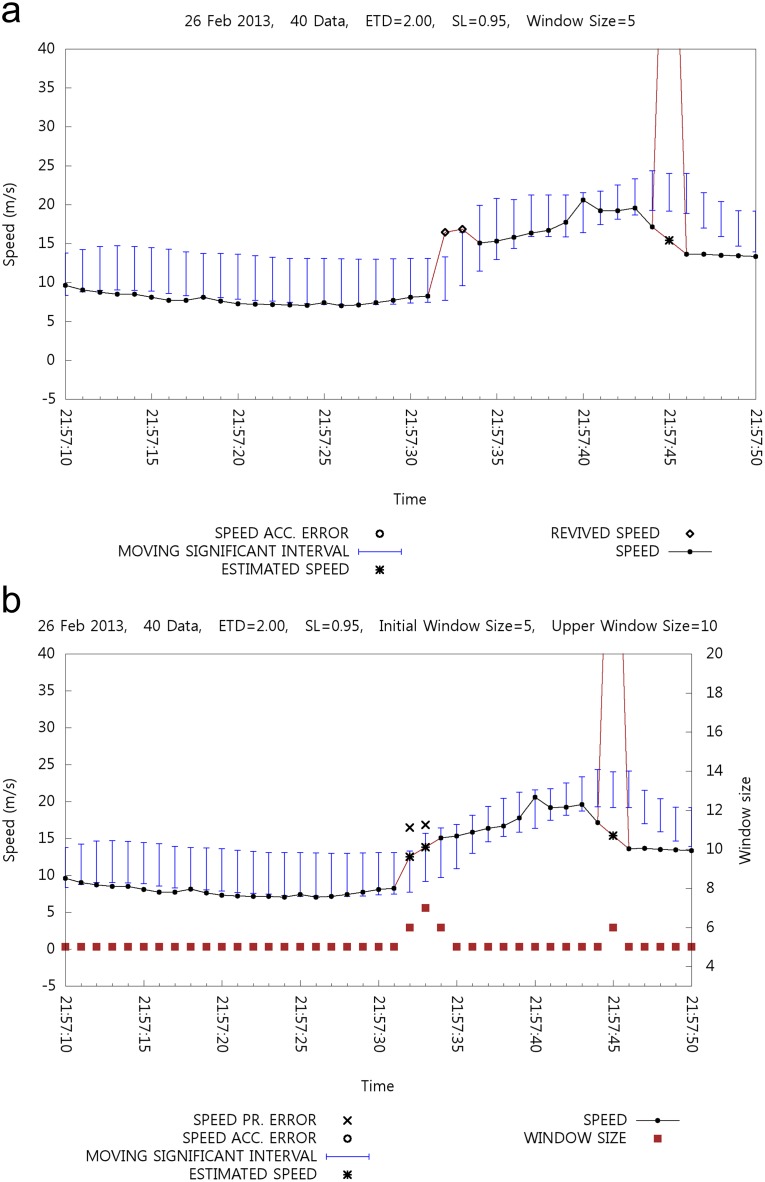
Case2: Effect of Window Size Adjustment.

Fig 17 shows another case where an error is restored with a fixed size window, whereas an error cannot be retrieved with a variable size window. It seems that the window size adjustment mechanism can successfully manage rapid increments of the MSI. With a fixed size window shown in Fig 17a, two errors are detected at 21:57:32 and 21:57:33 and then restored. Fig 17b shows the case of window size adjustment where the two retrieved speed data in Fig 17a cannot be retrieved in Fig 17b because of the stabilized MSI with a larger window size.

Simply speaking, window size adjustment is a mechanism to avoid underfiltering.

## Conclusion and Discussion

We developed an algorithm for detection and correction of positioning errors for portable devices. Based on the consecutive inputs of time and position values, speed and acceleration values can be calculated, and the detection of abnormal speed values can be performed based on moving windows. An unreal acceleration value is used for erroneous position and abnormal speed value detection. The most recent speed values and geographic positions are especially managed in a moving window. The moving window tracks the tendency of speed and position at a given time, and a moving significant interval is used to detect the outliers in the speed values. In order to manage moving window statistics, the characteristics of a positioning system are considered, and user-defined parameters, both dynamic and static, are identified and adjusted. We found that a small window size, such as ranging from five to ten, fits well for our purpose. In order to maintain moving window statistics and error correction, several mechanisms such as speed calibration, speed and position estimation, acceleration restriction, and backtracking were embedded in the algorithm. In addition, a dynamic window size adjustment mechanism was considered. All such mechanisms were tested through experiments for various aspects.

Our algorithm can be used with any equipment that has positioning facility. For example, our algorithm can be used for a GPS receiver with an extremely low memory and battery and CPU capacity. The algorithm seeks real-time detection of erroneous positioning data and pseudo real-time estimation of erroneous positioning data. The estimation of erroneous positioning data requires only one more positioning data be obtained. Along with the backtracking-revival mechanism, the determination for revival can be made after only two more positioning data are obtained.

More consideration is still required. In this paper, a probability distribution for human mobile speed was required. Several studies have deduced meaningful distributions [23–25], and from them exponential distribution was selected because of its simpler computation. The exponential distribution appears effective only for a limited speed range, i.e., while the speed range covers the usual human mobile speed.

Apart from human mobile speed distribution, the error in human mobile speed may also have a distribution. It is well known that the position error in <*latitude*, *longitude*> follows the bivariate normal distribution while the latitude and longitude are independent. The derivation of the distance between two positions became *χ* ^2^ distribution, and the derived speed has the same distribution. With window size *n*, the speed distribution in a moving window is *t*− distribution with *n* degrees of freedom. Clarifying the speed distribution in a moving window theoretically is required. This could be an open problem in the area of statistics.

Our next topic is the verification of the moving window statistics with the theory of probability distribution. In other words, we must have either the distribution of human mobile speed or the distribution of speed error to implement the algorithm presented. The distribution of the human mobile speed was utilized in this paper. The user-defined parameter of significant level, *s*, can be used experimentally from our results. There may be another possible implementation of our algorithm with the probability distribution of speed error for human mobile speed, although the distribution is far more complicated when compared with the exponential distribution that we introduced.
